# Factors influencing the performance of standard precautions for healthcare-associated infections by the nurses in intensive care units

**DOI:** 10.1186/s12912-025-02874-8

**Published:** 2025-03-04

**Authors:** Naryeong Kim, Minkyung Gu, Youngmi Cho, Oksun Kim, Sohyune Sok

**Affiliations:** 1 Department of Nursing, College of Nursing Science, Graduate School, Kyung Hee University, 26, Kyungheedae-Ro, Dongdaemun-Gu, Seoul, 02447 Republic of Korea; 2 Department of Nursing, College of Health Science, Daejin University, Pocheon-Si, Gyeonggi-Do, Republic of Korea; 3 Department of Nursing, Sun Moon University, Asan-Si, Chungcheongnam-Do 31460 Republic of Korea; 4 Department of Nursing, Kyungbuk Collge, Yeongju-si, Gyeongsangbuk-Do Republic of Korea

**Keywords:** Standard precautions, Performance, Infection, Nurse, Intensive care unit

## Abstract

**Background:**

In intensive care units, nurses’ management of healthcare-related infections is an important task that affects patient recovery, length of hospitalization, and medical costs. This study aimed to examine the relationships among health beliefs, knowledge, and performance related to standard precautions, and the factors influencing performance of standard precautions of intensive care unit nurses.

**Methods:**

A cross-sectional descriptive design was employed. Participants were 113 nurses with three months or over of work experience in the intensive care units (ICU) at the K University Hospital in South Korea. Measures were the general characteristics list, the health beliefs scale, the knowledge scale, and the performance scale of standard precautions. Data were collected from October to December, 2023. Data were analyzed using SPSS PC + version 29.0 statistical software program.

**Results:**

The health beliefs of standard precautions (β = 0.40, *P* < 0.001), work experience in the current department (β = 0.24, *P* = 0.019), and educational background (β = -0.23, *P* < 0.044) were statistically significant factors influencing performance of standard precautions of ICU nurses. The explanatory power of the final regression model was 47.0%.

**Conclusion:**

The health beliefs of standard precautions were important factor associated with performance of standard precautions of ICU nurses. Interventions strengthening health beliefs of standard precautions could improve performance of standard precautions. Increasing work experience in the current department and educational level need to be considered when developing and implementing interventions. ICU nurses should pay attention to factors influencing performance of standard precautions in hospital.

## Background

In the national policy on infectious disease, the importance of healthcare providers following standard precautions, such as hand hygiene and wearing personal protective equipment was emphasized [1, 2]. The focus was on those who are vulnerable to the risk of infection from air, droplets, blood, and bodily fluids from patient contact [3]. In the medical field, the intensive care unit (ICU) is especially a department where intensive treatment is performed on the patient. Also, the risk of infection increases significantly due to factors such as the patient’s underlying medical conditions, decreased immunity, and the severity of the disease [3]. The majority of healthcare-associated infections occurring in 5–10% of inpatients were centered in the ICU, in the order of urinary tract infections, pneumonia, surgical site infections, and bloodstream infections [4]. These healthcare-associated infections have delayed patients’ recovery and increased hospital stays, further increasing the burden of medical costs on patients and their caregivers [5]. Currently, in the adequacy assessment of the ICU in medical institutions in Korea, the performance score of the four BUNDLEs (a bundle of evidence-based control guidelines, such as hand hygiene practices and aseptic practice compliance of health care providers), which is one of the infection-related monitoring indicators, is relatively high, with a national average of 2.7 out of 3 points. However, a significant difference has been found in the results of these infection-related monitoring outcomes between general hospitals and long-term care facilities. Therefore, clear supervision and assessment of whether these infections are properly controlled in the ICU [6] must be performed.

Meanwhile, standard precautions are a concept developed by the Center for Control and Prevention (CDC) in the United States for common infections. They are infection control guidelines that must be followed to prevent exposure to secretions and excretions from all patients, such as blood, bodily fluids, and sputum [7]. They are also the most basic guidelines for all procedures, like the procedures for patients and the care of patients in medical institutions. Healthcare professionals must follow the standard precautions to ensure patient safety and prevent infection [5]. In particular, nurses account for the largest number of personnel in the medical institution and have the most frequent direct contact with patients in the actual medical field. Thus, it is imperative to comply with the standard precautions, which are the basic practices for fulfilling health beliefs [1, 4]. Health beliefs are concepts that describe health behaviors. These notions include the practice of maintaining and controlling behavior, which is the most fundamental element in the practice of care [8–10]. In other words, nurses must continue to strengthen infection prevention behaviors while complying with the standard precautions. Moreover, they must perform core nursing care based on health beliefs in care practices to reduce the occurrence of healthcare-associated infections [8, 11].

When examining the literature related to the performance of standard precautions, the performance of standard precautions of Korean nurses was 94.3% in general hospitals, 90.8% in small and medium-sized hospitals [12, 13], and 67.1% in public medical institutions [11, 14], and only about 46% of overseas nurses reported that they followed the standard precautions guidelines when performing invasive treatments [15]. In addition, while there were research results that reported knowledge of standard precautions as a factor that significantly affected performance of standard precautions [16, 17], there were also research results that reported that knowledge and health beliefs did not have much of an effect [14, 18]. These conflicting results of previous studies make it difficult to generalize the relationships among related knowledge, health beliefs, and performance of standard precautions, and therefore, repeated research is needed.

Knowledge related to health beliefs about standard precautions will play an important role in implementing standard precautions for healthcare-associated infections control by intensive care unit nurses [19]. Health beliefs and knowledge about standard precautions are especially important for intensive care unit nurses who perform infection control nursing in cooperation with many healthcare organization personnel [20, 21]. Intensive care unit nurses should actively implement standard precautions to increase the therapeutic effect of patients and achieve nursing performance related to infection [20, 21]. This study will be able to provide basic data necessary to improve the quality of nursing care for infection control by nurses working in intensive care units in Korea and to seek management measures for healthcare-associated infections occurring in intensive care units.

The purpose of this study was to confirm health beliefs, knowledge, and performance related to standard precautions among ICU nurses and to analyze factors influencing performance of standard precautions. The specific aims were to (1) identify the general characteristics of ICU nurses, (2) examine the levels of health beliefs, knowledge, and performance of standard precautions of ICU nurses, (3) identify the items with the highest level of performance of standard precautions for ICU nurses in order, (4) examine the correlations between health beliefs, knowledge, and performance of standard precautions of ICU nurses, (5) examine the factors influencing performance of standard precautions by ICU nurses.

## Methods

### Design, setting and participants

A cross-sectional descriptive design was employed. The inclusion criteria for study participants was nurses in the ICU of a general hospital in Seoul, South Korea. Exclusion criteria included managers of nursing units and nurses with less than three months of work experience because it was concluded difficult for them to directly perform nursing duties. The general hospital where this study was conducted has 3 (general, emergency, neonatal) ICUs, an average of 40 nurses working in 1 ICU, and the average infection rate of patients in ICU is 8.33%. In terms of physical structure, alcohol dispensers are readily available in all treatment rooms used by medical personnel (doctors, nurses), etc. At the same time, they are installed in patient rooms, nurses’ stations, and restrooms. At least one washbasin is installed in every room (treatment room, patient room, etc.) in the hospital. At this general hospital, standard precaution training is provided once when a nurse is first hired, and the training is provided irregularly thereafter as needed.

The number of participants was calculated with a significance level of 0.05 and an effect size (r) of 0.15 to obtain a statistical power (1-β) of 80% for the regression analysis of the G*Power 3.1.5 program [19]. Therefore, the appropriate sample size was 105 people, and the questionnaire was distributed to 126 people, considering the dropout rate of approximately 20% of the questionnaire. Out of 126 questionnaires in this study, 113 questionnaires were recovered, excluding five incomplete questionnaires and eight unrecovered questionnaires, which were finally used in the statistical analysis of this study.

### Instruments

A set of general characteristic variables consisted of a total of 7 items including gender, age, educational level, total clinical career, current department, current departmental career, and participation of standard precautions education.

To assess performance of the standard precautions, the scale developed by Hong et al. [16] based on the standard precautions of Korea Centers for Disease Control and Prevention (KCDCP) was used. This scale utilizes 36 questions, such as “Wash your hands with soap and water or use hand sanitizer even if there are no visible contaminants,” “Perform hand hygiene practice before wearing gloves,” and “Wear a mask when there is a possibility of contact with blood, bodily fluids, or secretions.” Each question has a five-point Likert scale, ranging from “not performing at all” to “performing very well,” with higher scores indicating higher performance of standard precautions.

To assess health beliefs of standard precautions, the scale developed by Erkin and Ozsoy [20] was modified and complemented by Ryu [21] to conform to the standard precautions. This scale has 29 questions—eight questions of perceived sensitivity, four questions of perceived severity, six questions of perceived benefit, eight questions of perceived disability, and three questions of behavioral triggers. Each question consists of “Healthcare-associated infections can occur even if the patient is not susceptible to infection, such as immunocompromised patients,” “Healthcare-associated infections will interfere with daily activities,” and “Performance of standard precautions will prevent cross-infection among patients.” This scale is based on a five-point Likert scale with higher scores of questions indicating higher health beliefs of standard precautions.

To measure the knowledge of standard precautions, the standard precautions knowledge scale that Cho and Choi [22] developed for the nurses in the ICU and emergency departments was used. It consists of 20 questions, such as “The most emphasized part of standard precautions is hand hygiene with alcohol,” “Gloves should be worn when touching objects contaminated with blood,” and “Care should be taken not to contaminate clothing, skin, and mucous membranes with contaminated linen, and be careful not to contaminate others.” The questions comprised of “yes” and “no”, where “yes” was scored as 1 point and “no” as 0 point. The higher the score of the question, the higher the knowledge of the standard precautions.

### Data collection

Data were collected from October to December, 2023. We contacted the hospital nursing unit manager and preliminary study participants to request for cooperation and conduct the study. Study participants were provided with a detailed information and a consent form for this study. A researcher directly distributed the self-report questionnaires and collected the completed questionnaires from the collection box. The entire survey took about 20 to 25 min to complete. After the questionnaire was completed, a small gift was provided to all study participants.

### Ethical considerations

This study was conducted after receiving approval from the Institute Research Review of K University, Seoul, South Korea (IRB No. KHSIRB-23-371-RA). First, the researcher visited the hospital nursing unit manager and received the necessary permission to conduct the study. The purpose of the study, provision of personal information, anonymity, and confidentiality were explained to all nurses participating in this study. Study participants were informed that they could withdraw from the study at any time if they wished to discontinue participation in the study, and it was explained that there would be no disadvantages to doing so. To ensure the safety of data collection, all information used was anonymous, and the completed questionnaire was sealed in a return envelope.

### Data analysis

The collected data were analyzed using SPSS Window version 29.0 program (IBM Corp., Armonk, NY, USA). General characteristics of the study participants were analyzed by frequency and percentage using descriptive statistics. The levels of the study variables were analyzed using descriptive statistics and frequency analysis with mean and standard deviation values. The order of high performance of standard precautions was analyzed using t-test. The correlations between study variables were analyzed using Pearson’s correlation coefficient. Factors influencing performance of standard precautions were analyzed using multiple linear regression of the stepwise method (dummy-coded variables were utilized). Independent variables were general characteristics of study participants (gender, age, level of education, total clinical career, current department, current departmental career, and participation of standard precautions education) and study variables (health beliefs and knowledge). Dependent variable was performance of standard precautions. For all analysis, a two-tailed *p* < 0.05 set as the significance level.

## Results

### Demographic and general characteristics of study participants

Table 1 shows demographic and other data of study participants in this study. The study subjects were a total of 113 nurses, and they included 102 women (90.3%) and 11 men (9.7%). In terms of age, 63 subjects (55.8%) were between the ages of 25 and 29, and the average age was 29.91 years. In terms of educational level, 102 subjects (90.3%) graduated from four-year colleges, and 60 subjects (53.1%) had less than five years of clinical experience. Currently, the general ICU accounted for the largest number of nurses with 53 subjects (46.9%), followed by the emergency ICU with 38 subjects (33.6%) and the neonatal ICU with 22 subjects (19.5%). In this study, 68 subjects (60.2%) had less than two years of experience in their current department, and 102 subjects (90.3%) had received training on standard precautions (Table 1).

**Table 1:** Demographic and general characteristics of study participants

Characteristics	Frequency		Percentage	Mean (SD)
Gender				
Female	102	90.3		29.91 (8.12)
Male	11	9.7		
Age (year)				
< 25	15	13.3		
25–29	63	55.8		
30–34	15	13.3		
35–39	7	6.2		
≤ 45	13	11.4		
Educational level				
College	2	1.8		
University	102	90.3		
≥ Master	9	7.9		
Total clinical career (year)				
< 5	60	53.1		
5–9	28	24.9		
10–14	10	8.8		
15–19	5	4.4		
20 ≤	10	8.8		
Current department				
General ICU	53	46.9		
Emergency ICU	38	33.6		
Neonatal ICU	22	19.5		
Current departmental career (year)				
< 2	68	60.2		
2–4	23	20.4		
5–9	13	11.4		
10 ≤	9	8.0		
Participation of standard precautions education				
Yes	102	90.3		
No	11	9.7		

### Levels of health beliefs, knowledge, and performance of standard precautions

The possible score for the performance of standard precautions was 36 to 180 points, with a median score of 174 and a mean score of 168.41 (12.95) points. The possible score for the health beliefs of standard precautions was 29 to 145 points, with a median score of 102 points and a mean score of 102.60 (8.20) points. The possible score for the knowledge of standard precautions was 0 to 20 points, with a median of 20 points and a mean score of 19.22 (1.10) points. Most mean scores of the health beliefs and knowledge were similar to the median scores (Table 2).

**Table 2:** Levels of health beliefs, knowledge, and performance of standard precautions

Variables	Possible scores	Median	Min	Max	Mean (SD)
Performance of standard precautions	36–180	174.00	140.00	180.00	168.41 (12.95)
Health beliefs	29–145	102.00	84.00	122.00	102.60 (8.20)
Knowledge	0–20	20.00	15.00	20.00	19.22 (1.10)

### Ranking of performance of standard precautions

Hand hygiene with alcohol was the highest at 46.27 points (SD 4.03), followed by wearing personal protective equipment at 41.73 points (SD 3.79), and safe injection at 23.99 points (SD 1.90). The lowest ranking was linen management at 9.30 points (SD 0.92), followed by hospital environment management at 9.30 points (SD 1.02), and medical supplies management at 9.43 points (SD 0.84) (Table 3).

**Table 3:** Ranking of performance of standard precautions

Performance of standard precautions			
Categories	Mean (SD)	t	Ranking
Hand hygiene with alcohol	46.27 (4.03)	122.08	1
Wear personal protective equipment	41.73 (3.79)	117.04	2
Safe injection practices	23.99 (1.90)	134.45	3
Respiratory etiquette	14.00 (1.33)	111.93	4
Employee safety	13.38 (1.19)	128.45	5
Medical supplies management	9.43 (0.84)	118.84	6
Hospital environment management	9.30 (1.02)	97.25	7
Hospital linen management	9.30 (0.92)	108.05	8

### Correlations between performance of standard precautions and the study variables

The correlation between the performance of standard precautions and the health beliefs of standard precautions was found to be a positive correlation (*r* = 0.31, *p* < 0.001). On the other hand, there was no significant correlation between the performance of standard precautions and knowledge of standard precautions, and between the health beliefs of standard precautions and the knowledge of standard precautions (Table 4).

**Table 4:** Correlations between performance of standard precautions and the study variables

Variables	Performance of standard precautions	Health beliefs	Knowledge
	r (*p*)		
Performance of standard precautions	1		
Health beliefs	0.31 (< 0.001^*^)	1	
Knowledge	0.02 (0.844)	0.42 (0.660)	1

^*^p < 0.05

### Factors influencing performance of standard precautions

The regression model with general characteristics was statistically significant (F = 3.22, *p* < 0.001). The statistically significant variables in this study were health beliefs of standard precautions (β = 0.40, *p* < 0.001), work experience in the current department (β = 0.24, *p* = 0.019), and educational level (β = -0.23, *p* = 0.044), and the explanatory power of the final regression model was 47.0% (Table 5).

**Table 5:** Factors influencing performance of standard precautions

Variables	B	S.E	β	*t*	*p*	95% CI	
						Lower	Upper
Gender	0.39	0.38	0.16	1.03	0.306	-0.36	1.14
Age (year)	3.97	3.50	0.10	1.13	0.260	-2.98	10.92
Educational level	-10.12	4.97	-0.23	-2.04	0.044^*^	-19.97	-0.26
Total clinical career (year)	-0.19	0.02	-0.15	-1.02	0.310	-0.56	0.02
Current department	0.62	1.63	0.04	0.38	0.704	-2.62	3.86
Current departmental career (year)	0.10	0,04	0.24	2.39	0.019^*^	0.17	1.79
Participation of standard precautions education	-3.98	4.11	-0.09	-0.97	0.335	-12.13	4.17
Health beliefs	0.62	0.15	0.40	4.31	< 0.001^*^	0.34	0.91
Knowledge	-1.34	1.06	-0.11	-1.27	0.205	-3.44	0.75
Adj. R^2^ = 0.47, F = 3.22, *p* < 0.001^*^							

*B* Unstandardized coefficients, *S.E.* Standard Error, *β* Standardized coefficients, *t* t test, *CI* Confidence Interval, *Adj. R*^*2*^ Adjust R-squared, *F* F test

^*^p < 0.05

## Discussion

The mean score for knowledge related to the standard precautions was 19.22 out of 20 points, which can be converted into a mean percentage of a high level at 96.1%. This result is in line with Kim and Park’s findings [13], which examined hospital nurses’ knowledge of standard precautions, nursing intuitions, and infection control organizational culture. The global pandemic of infectious diseases such as COVID-19 has further strengthened the standard precautions of medical institutions regarding the previous infection control guidelines and epidemic prevention rules. The study’s finding suggests that the knowledge level of healthcare providers has increased even further through national and institutional support in preventing infectious diseases. In this regard, ICU nurses need to be knowledgeable of the standard precautions related to new infectious diseases and actively modify and enhance the standard precautions against infectious diseases. In particular, ICU nurses must be able to accurately and quickly implement standard precautions to control healthcare-associated infections [23].

In this study, the standard precautions were performed most frequently in the order of hand hygiene with alcohol, wearing of personal protective equipment, and safe injections, whereas they were the least performed in the order of the management of medical treatment supplies, hospital environment, and linen. In the actual medical field, especially in the ICU, most medical assistants, such as nursing assistants, are in charge of disinfection or re-arranging medical treatment supplies and linen used for patients. This finding implies that ICU nurses may lack knowledge in managing medical treatment supplies or hospital environments [24]. Nurses are responsible for managing the hospital environment, medical supplies, and supervising medical assistants, and they must have practical competence related to these tasks [24].

The correlation between the performance of standard precautions, health beliefs, and knowledge analyzed in the study resulted in a significant positive correlation between the performance of standard precautions and health beliefs. This supports the findings of Ryu [21], who examined the factors influencing the performance of standard precautions for clinical nurses. And the performance of standard precautions of ICU nurses were found to have no correlation with related knowledge or education level. This was similar to the results of previous studies [16, 23, 24]. It showed that having related knowledge does not necessarily mean that the knowledge is translated into practical behavior in clinical settings. However, previous studies have demonstrated conflicting results to some extent; hence, further study is required to generalize the results among the standard precautions, health beliefs, and knowledge [22]. Due to the prolonged epidemic, it is necessary to strengthen education on hospital infection control and continuously awaken the perceived sensitivity and motivation of the ICU nurses toward standard precautions through systematic education and training [23, 25, 26].

Finally, in this study, the factors influencing the performance of the ICU nurses were health beliefs of standard precautions, work experience in the current department, and educational background. This is supported by a study by Kim and Cha [27] on the performance of ICU nurses in multidrug-resistant infection control in Korean medical institutions, where the findings indicate that the perceived sensitivity of nursing personnel in their health beliefs to promote patient health can further increase the immediate therapeutic effect of patients and the benefits of their work. Therefore, it is imperative to develop a systematic job training program that can trigger action to increase the perceived sensitivity of ICU nurses to health beliefs and actively implement it in medical institutions [25, 26].

The above findings address the need to strengthen ICU nurses’ health beliefs to improve their standard precaution performance. Implementing systematic and continuous job training programs related to standard precautions from the perspective of improving perceived sensitivities to reinforce health beliefs is needed. However, the findings reveal that there is no relationship between the ICU nurses’ performance and knowledge of standard precautions. Accordingly, the nurses may not perform standard precautions even if they have sufficient knowledge, so the benefits should be considered in depth. Furthermore, even nurses knowledgeable about the correct standard precautions and proficient in nursing related to infections may adopt a passive work attitude over time due to the repeated emotional labor of caring for patients with infectious diseases [28]. To reduce the passive work attitudes of ICU nurses toward health beliefs and knowledge of standard precautions, healthcare institutions should develop in-house processes and provide the conditions required to create a comfortable working environment [25, 26]. In addition, by providing internal and external stimuli, such as rewarding departments or employees with high rates of standard precautions performance and disseminating these rewards to other employees, nursing managers can have a positive effect on improving the standard precautions performance of ICU nurses. It is also vital to render efforts to specifically modify and improve standard precautions in medical institutions. Further study with the same context and subjects must be conducted to include various factors affecting the performance of standard precautions by ICU nurses, considering the characteristics of Korean hospitals. Also, there is a need for continuous research that will further develop the performance of standard precautions for each hospital department in South Korea and validate its feasibility.

### Limitations

Although there is a lack of relevant studies on the standard precautions performed by ICU nurses in South Korea in general, the current study only covered ICU nurses of a general hospital in Seoul. Such sampling limitation influences the complete justification of the aspects affecting the ICU nurses’ performance of the standard precautions in South Korea. Moreover, the failure to thoroughly consider the confounding variables, such as the characteristics of the hospital, the ICU environment, and the relationship with the ICU personnel may serve as a limitation of this study. Also, since nurses who were not observed using the performance evaluation scale were asked to self-report whether they performed these guidelines, the interpretation of findings may be limited accordingly. One additional limitation is the limited time period in which this survey was performed and may reflect sentiments that have changed. Thus, repeated and expanded studies considering the sampling of a more extensive population and the other influential contexts and settings, such as the features of the research site or hospital and the relationship of the ICU personnel and nurses, are recommended.

## Conclusion

This study demonstrated that the factors influencing performance of standard precautions were the health beliefs associated with the standard precautions, the work experience of the current department, and their educational levels. This study is significant as it identifies ICU nurses’ performance of standard precautions and measures the relationship between the health beliefs and knowledge related to them. Furthermore, it explored fundamental data that can help future researchers and healthcare sectors strategize to improve the job performance of ICU nurses in relation to infection control.

## Data Availability

No datasets were generated or analysed during the current study.
